# The promise of healthcare reform in transforming services for jail releasees and other criminal justice populations

**DOI:** 10.1186/2194-7899-2-9

**Published:** 2014-04-24

**Authors:** Maureen McDonnell, Laura Brookes, Arthur J Lurigio

**Affiliations:** 1 Center for Health and Justice, TASC, Inc.,, 1500 N Halsted Street, Chicago, IL 60640 USA; 2 College of Arts and Sciences, Loyola University Chicago, Sullivan Center Room 230, 1032 W. Sheridan Road, Chicago, IL 60660 USA

**Keywords:** Jails, Behavioral healthcare, Affordable care act, Detainees, Releasees, Drug treatment, Psychiatric treatment, Integrated care

## Abstract

Chronic behavioral health conditions, such as psychiatric and substance use disorders, affect at least half of all arrestees, with two-thirds suffering from at least one chronic medical disorder. These conditions contribute to their criminal behaviors and propensities to recycle through the criminal justice system (Binswanger et al. Journal of Urban Health 89:183-190, 2012). Despite their limited resources, jails have nonetheless become *de facto* settings for the delivery of healthcare services. With the passage of the Affordable Care Act (ACA) of 2010, jail releasees will become eligible for government-subsidized healthcare coverage in 2014. The widespread availability of integrated healthcare services for the released jail population is likely to reduce criminal behavior, which is often associated with psychiatric and substance use disorders and their co-occurrence. This article provides an overview of behavioral healthcare services available to jail releasees. We discuss the evolving landscape of substance use and mental health interventions under healthcare reform, including anticipated changes in funding infrastructures and streams for treatment services. We examine the financial and practical implications of these changes for the criminal justice system, particularly for the nation’s jails.

This article presents an overview of behavioral healthcare services for the justice-involved population. We discuss the evolving landscape of substance abuse and mental health interventions under healthcare reform, including anticipated changes in funding infrastructures and streams for treatment services. We examine the financial and practical implications of these changes for the criminal justice system, particularly for the nation’s jails. To realize the opportunities of healthcare reform, we recommend a vigorous and coordinated response from institutional and community-based treatment providers, government officials, Medicaid and insurance directors, and criminal justice administrators.

For the purposes of this article, the terms “justice-involved population”, “jail population, releasees”, and “former detainees”, are used interchangeably to mean individuals being released from jail custody and transitioned into the community. The “criminal justice system” refers to the broad system of courts, jails, and probation departments within a county. This article is geared toward jurisdictions that operate jails—whether small, medium, or large—as nearly all jails are heavily impacted by populations with untreated substance use disorders and serious mental illness. Although individuals who have been convicted and sentenced are ineligible to receive Medicaid benefits while they are in custody, federal policy states that people detained in jails, while still detained, are eligible to apply to receive Medicaid benefits after their release (Medicaid Program; Eligibility Changes Under the Affordable Care Act of 2010, 2012).

## Background

### Jails as healthcare settings

Approximately 12 million adults churn through jails each year in the United States (Milton 2012). Chronic health conditions, such as psychiatric and substance use disorders, affect at least half of all arrestees and contribute to their criminal behaviors and propensities to recycle through the criminal justice system (Office of National Drug Control Policy [ONDCP] 2009). As the front door to the criminal justice system, jails are one of the largest catchment areas for people with behavioral healthcare problems, infectious diseases, and serious medical conditions. In addition, a substantial majority of detainees enter jail having no private or public health insurance, especially in states that exclude childless adults from Medicaid eligibility. Hence, detainees’ disorders are typically under-treated or not treated at all following their release into the community (Regenstein & Maples-Christie 2012).

The primary goals of the criminal justice system are to protect public safety and lower recidivism through rehabilitative programming and services. From a philosophical, structural, administrative, funding, and human resources standpoint, criminal justice agencies are ill equipped to provide healthcare services and often do so only under the threat of litigation (Harris & Lurigio 2009). Indeed, case precedents and constitutional safeguards have established the right of individuals in jails and prisons to receive medical care that attends appropriately to their needs within each medical specialty. For example, in reference to the Texas prison system, *Estelle v. Gamble* determined that “deliberate indifference” to the healthcare needs of inmates constituted a violation of 8th Amendment protections against cruel and unusual punishment. Case law regarding the right to treatment in jail settings has also cited 14th Amendment due process protections for pretrial detainees. Correctional institutions have struggled to carry out these mandates (Manderscheid et al. 2004).

Wide-scale diversion and jail reentry programs are unavailable in most jurisdictions, and most community and behavioral healthcare systems lack the services to meet all the treatment needs of the jail detainees, 90 percent of whom are uninsured (Wang et al. 2008). Current procedures for enrolling in Medicaid and maintaining benefits eligibility are burdensome and inefficient and unnecessarily preclude many eligible individuals from obtaining services, especially those with psychiatric disorders (Morrissey et al. 2006). Furthermore, jail-based healthcare is rarely integrated with community-based healthcare, thereby resulting in discontinuities in the recovery process before, during, and after detention while also contributing to relapses, recidivism, and visits to emergency departments (Veysey et al. 1997).

### Affordable care act

President Barack Obama signed the Patient Protection and Affordable Care Act (P.L. 111-148) and the Health Care and Education Reconciliation Act of 2010 (P.L. 111-152) into law in March 2010. This reform initiated significant change in the country’s healthcare system and in the regulation of its healthcare insurers. Known as the Affordable Care Act (ACA), the primary goals of this reform are to reduce the overall costs of healthcare and enhance the quality, coordination, and accessibility of healthcare services, particularly among people who have been uninsured because of preexisting conditions. The goals of the ACA will be achieved using subsidies, mandates, and tax credits that foster broader healthcare coverage among individual insurance carriers and employers (Congressional Budget Office [CBO] 2012).

Beyond the impact of this legislation on healthcare services for the American people as a whole, the most vulnerable populations are now able to receive general and behavioral healthcare services for the first time in their lives. In many states, healthcare exchanges, low-income health subsidies, and Medicaid expansion afford services to individuals who have been denied equitable healthcare opportunities because of gross economic disparities (Regenstein & Maples-Christie 2012).

Jails have become *de facto* settings for the delivery of healthcare services among disproportionally low-income populations. To date, the majority of this population has relied on specialized federal funding. With limited funding in the community for medical, substance abuse and mental health treatment for indigent men and women, the majority of this population has relied on federal, state and local funding for the uninsured, such as funding provided for hospital-based charity care, community health centers, and public substance abuse and mental health treatment. Federal agencies such as the Substance Abuse and Mental Health Services Administration and the Bureau of Justice Assistance have dedicated grant-making portfolios that focus on improving care for people under justice supervision, including those leaving jails. Particular pieces of federal legislation have expanded both resource and model development, including the 2008 Second Chance Act and the 2008 Mentally Ill Offender Treatment and Crime Reduction Act, specifically for behavioral health treatment only, triggered by a person’s involvement in the justice system (P.L. 110-416).

With the enactment of the ACA in January 2014, many low-income jail releasees have become eligible for government-subsidized healthcare coverage. The widespread availability of integrated behavioral healthcare services for this population is likely to reduce criminal behavior, which is often associated with psychiatric and substance use disorders and their co-occurrence (Council of State Governments Justice Center [CSGJC] 2013). Moreover, the ACA is expected to generate cost savings for county and state governments by expanding effective reentry services and providing jail releasees alternatives to incarceration (Regenstein & Maples-Christie 2012).

The original intention of the ACA was to expand Medicaid eligibility nationwide to all single adults under the age of 65 whose annual income falls below 138 percent of the Federal Poverty Level (FPL) ($16,105 for individuals, $21,707 for couples) and mandate the purchase of insurance coverage to all those above this mark^a^. Eligibility for ACA subsidies also requires that applicants be (1) ineligible for Medicare, (2) United States citizens or legal residents for 5 years or more, and (3) in possession of a Social Security number or in the process of applying for one. In every state, low-income adults without children would also be guaranteed Medicaid coverage without a waiver, and parents of children would be eligible at the same income level.

The broad-scale implementation of the ACA was greatly affected by the Supreme Court case *National Federation of Independent Business v. Sebelius*, which challenged the constitutionality of both the individual mandate and the terms of state Medicaid expansion. In June 2012, the Supreme Court found that the penalties imposed on states for nonadherence to expansion were unconstitutional. Consequently, individual states can now elect to refuse participation in the ACA mandates without reducing their federal Medicaid assistance. Because of this ruling, nearly half of the states are expected to opt out of full expansion—at least in the first year (National Conference of State Legislatures 2013).

The same ruling, however, upheld the individual mandate to purchase insurance. Although a large proportion of jail detainees fall under the 138 percent FPL requirement, criminal justice systems in each state have differential access to the resources necessary to address releasees’ behavioral healthcare needs. Those participating in the expansion can draw upon Medicaid benefits for rehabilitation purposes to treat the criminogenic needs of detainees being released from custody. Notwithstanding the potential of the ACA to foster recovery and desistance from crime, the legislation might be limited in many states and, therefore, unable to alleviate significantly the serious healthcare problems of criminally involved individuals who typically require the comprehensive and coordinated medical and psychiatric care needed to achieve long-term sobriety and stability.

### Benefits of behavioral healthcare for jail populations

Treatment for behavioral healthcare problems can reduce crime and recidivism (Lurigio 2000; Messina et al. 2004). Still, the vast majority of arrestees with psychiatric and substance use disorders receive no treatment in either the community or correctional settings (Minton 2013; ONDCP 2009; Wilson 2000). As we noted above, basic healthcare in jails is rarely coordinated with community-based healthcare at intake or upon discharge from confinement (Solomon et al. 2008). The brief nature of jail stays also makes it difficult to deliver effective dosages of treatment during detention. In addition, the lack of insurance for jail releasees results in intermittent and inadequate care, which is primarily delivered in emergency departments.

Without jail-based prevention, early intervention, and treatment services for infectious diseases, releasees might return home with communicable illnesses (e.g., tuberculosis, Hepatitis C, and HIV), which increases health risks to partners, children, parents, extended families, and neighbors. Likewise, when care for psychiatric and substance use disorders is absent, inadequate, or interrupted in jails, these conditions persist within the detention population and result in the continued use of alcohol and drugs. This persistence subsequently results in the perpetuation of criminal activity, emergency department visits, intimate partner violence, child abuse and neglect, and DUI injuries and mortalities (Mueser & Drake 2007; Veysey et al. 1997). Furthermore, given the significant overrepresentation of African Americans in the correctional population, healthcare reform could decrease the health and justice disparities that stem from racial and socioeconomic inequalities (Mayberry et al. 2000) as well as problems of social disorder, economic decline, violent crime, and high rates of incarceration that are pervasive in urban communities (Hagan 2010).

Service delays and interruptions render health problems even more difficult and expensive to treat. Without coordinated care for the jail population, the criminal behaviors associated with co-occurring substance use and psychiatric disorders are likely to continue, creating undue risk to public safety and placing an economic burden on the community. The widespread provision of healthcare services could eliminate long waiting lists for care, end the piecemeal use of grant dollars to treat criminal justice populations, break the cycle of repeated criminal justice involvement, and reduce the country’s jail population—all without compromising public safety. The correctional dollars saved though the provision of near-universal healthcare could be reinvested to revitalize impoverished communities through enhancements in education, job training, and housing. In short, under the ACA, the near-universal eligibility for healthcare insurance among states that opt into Medicaid expansion could transform the delivery of publicly funded primary care, substance abuse, and mental healthcare services. As we discuss in the following section, many of these changes will affect service systems and their staffs.

### Healthcare reform and the behavioral healthcare systems

#### Capacity expansion and adaptation

The extent to which the costs of psychiatric and substance use services will be reimbursed under Medicaid or subsidized by private plans after the implementation of the ACA is unknown. The community-based treatment system must be prepared for a dramatic increase in the number of patients with government-subsidized insurance, including those in or coming out of jail. Hence, providers must decide when and how much to broaden their services even as they grapple with reductions in state funding. Large providers with greater reserves can build more substantial structures of services until the number of clients increase. Conversely, small providers are less likely to have this option and might miss these expansion opportunities.

#### Shifts in treatment modalities

The cost containment strategies used with the Medicaid program will focus on greater individualization of treatment applying the criteria of medical necessity. These criteria might result in an increase in the use of lower-cost modalities. Indeed, cutbacks in long-term care occurred in the private treatment system when managed care models became prevalent in the 1990s (National Association of State Alcohol and Drug Abuse Directors [NASADAD] 2010). Individualized care plans using all modalities in the system enable each patient to obtain the right treatment at the right time, especially during the most acute phase of an illness. Such effective management of illness is most likely to reduce chronicity and foster recovery. When managed care leads to managed recovery, consumers and their families benefit (Sommers et al. 2012). Thus, to sustain the full continuum of care needed to build recovery in the community, leaders in the treatment and criminal justice systems must be informed about changes in treatment venues and prepared to respond quickly to these changes as the implementation of the ACA unfolds.

#### Medicaid billing rules and requirements

Many people receiving care in the publicly funded treatment system will have health insurance coverage through the Medicaid program; its funding rules will govern how care is delivered, reviewed, and approved. In general, each participating state’s Medicaid authority will become a primary funder and serve as the oversight manager for services. Providers will likely operate in an environment, whether fee-for-service or managed care, where their services must meet medical necessity criteria (see next section). Providers will also be required to employ staff members whose credentials meet professional standards (NASADAD 2010).

The Medicaid billing environment will change significantly for publically supported substance abuse and mental health treatment services, which are largely underwritten by federal block grants and dedicated state and local funding streams that help maintain the treatment infrastructure in underserved communities (Jost & Rosenbaum 2012). Medicaid billing and verification requirements are fundamentally different from those in a block grant system and require authorization for all substance use treatment services. Under the new system, smaller and less sophisticated providers will be required to implement Medicaid-compatible fee-for-service billing protocols and preserve electronic records for use and documentation review.

#### Medical necessity criteria

Access to care through the Medicaid program is currently governed by the criteria of medical necessity. As customarily applied, urgently needed care is more likely to be reimbursed than long-term care (e.g., psychosocial rehabilitation). Substance abuse and mental healthcare providers, in particular, will need to examine the structure and funding of vital elements of treatment. If the federal block grant program is reduced, a major fiscal resource for supporting long-term care will become unavailable. For example, Massachusetts enacted a Medicaid expansion program that covers pre-authorized treatment services but excludes the housing costs associated with residential treatment (NASADAD 2010).

With the emphasis of healthcare reform on the criteria of medical necessity and the adoption of evidence-based practices, the use of medications in behavioral healthcare treatment is likely to rise. For example, the Substance Abuse and Mental Health Services Administration (SAMHSA) and the National Institute on Drug Abuse (NIDA) recognize medication-assisted treatment for addiction as an evidence-based practice (Friedmann & Schwartz 2012; NIDA 2012). Several new medications that decrease drug cravings and encourage recovery have been marketed in the past decade, particularly for the treatment of alcohol and opiate dependence (e.g., Weiss et al. 2011). Community treatment providers and criminal justice practitioners are beginning to incorporate such medications into their treatment regimes, whereas others oppose the use of medication and favor abstinence-only interventions (Knudsen et al. 2010). To partake fully in the healthcare options that will be reimbursable under the ACA, local providers might be compelled to revise their policies regarding medication-assisted treatment, particularly in light of research that demonstrates the positive impact of these medications on client success.

#### Workforce issues

As the demand for services increases under healthcare reform, substance abuse and mental healthcare providers can expect to encounter unprecedented workforce challenges and possibilities. In fact, the substance use treatment workforce is already facing a shortage of credentialed clinicians (Whitter 2006). In addition, behavioral healthcare providers will need to be trained in Medicaid procedures, which will entail learning new healthcare terminologies and reimbursement requirements, as well as more sophisticated information technology skills.

Practitioners in medical specialties also must plan for the possibility that relatively few healthcare providers and medical doctors, especially those practicing in less-populated suburban and rural areas, will be willing to serve difficult-to-treat, justice-involved consumers, particularly given the relatively low Medicaid reimbursement rates. On the other hand, improved care will become available by expanding resident training programs in primary and behavioral healthcare programs in underserved communities. In addition, the expansion of telemedicine—the delivery of healthcare services via video technology and interactive communication networks—will broaden the professionals’ reach into remote and underserved areas (Berman & Fenaughty 2005).

#### Federally qualified health centers

Under the ACA, primary healthcare for low-income people will be expanded and delivered through the existing network of Federally Qualified Health Centers (FQHCs). With healthcare reform, FQHCs might also provide more mental health and substance use services, especially low-intensity, outpatient, and medication-assisted treatment. Analogous to other chronic medical conditions, addiction, and mental health problems require ongoing, long-term treatment and case management services. As a result, better integration of primary and specialty care will lead to better service coordination, fewer discrete acute-care episodes over a lifetime, and more favorable patient outcomes. Service integration will benefit patients in the community regardless of their past justice system involvement; however, the potential health benefits and expenditure reductions are likely to be even greater for members of the justice-involved population, who are affected by high rates of infectious (e.g., HIV, tuberculosis, and hepatitis) and chronic diseases (e.g., psychiatric and substance use disorders). Thus, providers should attempt to create partnerships with FQHCs that could serve the justice-involved population by combining continuous coordinated care in the community with jail-based treatment for the justice-involved population moving into and out of confinement.

#### Regional concerns

In large metropolitan areas, primary and behavioral healthcare services are often located within the public healthcare sector, where providers compete with one another for clients and funding (Elmendorf 2009); nonetheless, providers can collaborate to construct a continuum of care to treat particular populations and diseases. In small cities and rural communities, typically only one or two providers serve a large geographic area (Fiscella 2011). Such providers must rely on block grant funding and will likely be among those most challenged by billing requirements and demands to offer services proactively with the expanded Medicaid program.

These rural providers also will be challenged to meet the client selection criteria of Medicaid given limited services and smaller populations. The market alone could never bear the full range of services needed to treat the jail population, especially in rural areas. Government officials must be prepared to play a leading role in the regional and state planning of primary healthcare, specialty substance use treatment, and psychiatric services. As a major referral source for publicly funded treatment, criminal justice agencies must also be fully engaged in this planning process.

#### Medicaid program expansion

The NASADAD (2010) examined the healthcare reforms instituted in Massachusetts, Maine, and Vermont. In these states, expansion resulted in substantially greater use of treatment, ranging from a 20 percent increase in Massachusetts (2006-2008), to a 32 percent increase in Maine (1999-2008), and a 100 percent increase in Vermont (1998-2007). The expansion of care enabled these states to integrate substance use treatment with primary healthcare services and provide more medication-assisted treatment options, especially for opiate dependence. Expansion also reoriented the systems away from the acute-care model toward a long-term recovery care model. NASADAD’s review also found that federal block grant funds were an important component of healthcare reform; these dollars cover non-medical (i.e., non-Medicaid reimbursable) services that are critical to rehabilitation, such as housing and psychosocial support services.

#### Service parity

The state process for determining healthcare benefits will be influenced by budgetary imperatives that emphasize maximal cost containment over maximal effectiveness in care. However, prioritizing cost containment over cost-effectiveness can perpetuate problems in the public specialty treatment and other healthcare systems. Such problems include the fragmentation of acute care episodes, the disproportionate consumption of resources by people with the most serious and complex problems, and the failure to implement evidence-based practices due to funding limitations. The interpretation of parity under the Mental Health Parity and Addiction Equity Act of 2008 involves the provision of the highest quality and most effective care at the most manageable cost rather than uniformly inadequate care at the lowest cost (U.S. Department of Labor [DOL] 2010). Through the ACA, the expansion of resources for substance use, mental health, and primary care services will serve more people and create better models of care in the community (Sommers et al. 2012).

#### Areas for improvement

In the healthcare reform era, the framework for behavioral healthcare must shift from the episodic treatment of acute illnesses in the emergency department toward the long-term management of chronic diseases with rehabilitative support in the community. Individuals with substance use disorders generally recover over a period of 2 to 5 years. Each acute episode of treatment can support this trajectory but, in itself, is insufficient to “cure” patients of their substance use disorders. The concepts of recovery management and recovery-oriented systems of care for addiction promote sustained sobriety, not simply the cessation of use, and include extensive formal and informal recovery support mechanisms (Lurigio et al. 2008; Scott et al. 2005). Broad adoption of these frameworks would likely improve outcomes and contain costs.

Individuals under criminal justice supervision often have numerous and complicated health problems (Peters & Petrila 2004). For this population, the newly formed healthcare system will improve the accessibility and integration of primary healthcare, substance abuse treatment, and mental health services. When care is delivered in septe systems, the likelihood of access to any of these services is diminished, and individuals are less likely to enroll in a sustained treatment program. Thus, the successful integration of all components of care is a priority under the ACA as it can foster access to services, enhance the clinical integration of all components of care, and lead to better treatment outcomes. An example of integrated care is the medical home model, which is being developed in the public primary care, mental health, and substance abuse treatment systems.

As in all branches of medicine, a number of proven protocols for services in the field of behavioral healthcare are underused, including interventions that are effective in treating criminal justice-involved individuals with substance use and psychiatric disorders (NIDA 2010). For example, SAMHSA’s *National Registry of Evidence-based Programs and Practices* includes more than 160 proven interventions in the prevention and treatment of psychiatric and substance use disorders and their co-occurrence. The implementation of the ACA and its changes in healthcare services and funding will likely encourage the adoption of evidence-based treatment strategies. Therefore, a coordinated effort to provide evidence-based practices with the implementation of healthcare reform will certainly increase treatment effectiveness on a broad scale.

The presence of serious trauma should also be assessed in correctional populations. Histories of serious trauma are common among people involved in the criminal justice system, especially women (Kubiak & Rose 2007). In addition, a history of trauma is associated with high rates of psychiatric and substance use disorders and their co-occurrence (Solomon et al. 2008). Furthermore, the attainment of sobriety often exposes underlying trauma that must be addressed to achieve long-term recovery (Kubiak 2004). To date, funding limitations have made trauma recovery services scarce for low-income individuals (Hamblen 2012). The ACA’s provisions will help remedy this situation and reduce substance use relapse and criminal recidivism (Mallik-Kane & Visher 2008).

### Opportunities for the criminal justice system

#### Enhanced leadership

Most detainees spend a relatively short time in jail, constraining the range and duration of jail interventions (Minton 2013). Nonetheless, criminal justice systems can play a pivotal role in reducing recidivism, protecting public safety, and curtailing public expenditures through diversion and reentry programs that link releasees to community-based services. Criminal justice officials are also in a unique position to spearhead local healthcare planning initiatives. In many jurisdictions, sheriffs, judges, and other criminal justice leaders have convened stakeholder groups to increase the availability of substance use and mental health interventions (Berman & Feinblatt 2001). Over the past 30 years, these coalitions have initiated and implemented diversion and intervention programs throughout the criminal justice continuum. Such partnerships can help design and manage interventions to control crime without compromising public safety (Steadman & Naples 2005).

Criminal justice officials’ participation in planning local healthcare reform is critical to avoid barriers that can discourage the use of treatment and interrupt the continuity of care. For example, Medicaid plans in participating states will likely contain provisions that approve less-intensive treatment modalities (e.g., outpatient programming instead of residential treatment). Nevertheless, this emphasis could increase incarceration rates if judges are reluctant to mandate offenders to outpatient treatment programs. Experienced providers must educate judges to ensure that comprehensive and integrated outpatient supervision and treatment plans are viewed as viable pretrial supervision and sentencing options. In addition, the Medicaid enrollment process must be simplified and streamlined. Releasees with psychiatric and substance use disorders are usually unable to navigate the complex decision-making protocols necessary to render healthful choices about recovery and rehabilitation. For this population, unfamiliar insurance enrollment and eligibility maintenance procedures will present significant and unexpected impediments to accessing and using healthcare services.

#### Improved services and cost-effectiveness

County governments will have a considerable stake in the success of the changing healthcare environment. These governments can benefit substantially from reduced incarceration costs, but they will bear the brunt of increased costs for jail-based medical care. They will also bear the brunt of potential litigation that could stem from the inadequate provision of healthcare services. County boards often control the funding for local criminal justice and court systems as well as for safety-net hospitals and public clinics; thus, their role is critical in reshaping the infrastructure of the county’s correctional systems to guarantee that jail releasees are placed in community-based healthcare programs and monitored effectively while receiving services.

Demonstration programs can transform the lives of participants; nonetheless, they rarely operate at full capacity or reach all individuals who are eligible for services. The lack of funding to expand successful models has limited the potential of the criminal justice system to reduce recidivism. Criminal justice officials are often frustrated by long waiting lists for placing clients into treatment and by the burdensome grant-writing process necessary to secure funding for even incremental increases in services. These circumstances are beginning to change with the implementation of Medicaid expansion, when nearly all people involved in the criminal justice system in participating states will be eligible for government-subsidized health insurance. Thus, the lack of funding for primary care services and the treatment of substance use and psychiatric disorders will no longer be a permanent barrier to implementing diversion and intervention services at each interception point in the criminal justice process. Most jurisdictions will have the option to bring the diversion and intervention programs already in place to the full scale as well as adopt other proven models of service (cf. Druss & Mauer 2010).

Investment in treatment and services in lieu of or in conjunction with criminal justice options can yield considerable savings to taxpayers who have carried the heavy financial burden as correctional populations have grown continually and precipitously over the past 30 years (NIDA 1999; Pew Center 2010). Front-end diversion or deflection strategies are the most cost-effective mechanisms for reducing preadjudication expenditures that arise from arrest, prosecution, and detention. These programs offer people with criminal involvement the opportunity to participate in services as an avenue to preclude further penetration into the system and begin (or continue) their recovery efforts.

To date, funding restrictions and short stays have kept jails from administering effective behavioral healthcare interventions for a large percentage of detainees and releasees (Regenstein & Maples-Christie 2012). As releasees become eligible for health insurance, criminal justice systems can use that funding to incorporate behavioral healthcare components in reentry programs that can help reduce repeated detentions. For example, if a midsize jail expands its deflection programs, including pretrial release with supervised community treatment, the county’s annual costs for detention services would reduce in proportion to the size of such programming. If these redirection programs resulted in only a 10 percent reduction in population size, a medium-sized facility could save more than $2 million dollars in annual detention costs^b^.

#### Shifting priorities

Healthcare reform might inadvertently increase the pressure on jails to deliver services as more detainees enter confinement in need of healthcare services initially received in the community. This responsibility could shift priorities in the criminal justice system from punishment, containment, and control to rehabilitation, treatment, and recovery. The high-stakes challenge is to design and implement a modified system that ensures improved and coordinated healthcare in the least-restrictive setting while upholding the protection of public safety.

Broad eligibility for insurance coverage could integrate care to a much greater extent than previously possible. Planning efforts should focus on enrolling detainees in Medicaid before their release from jail; transferring prescriptions from jails to community health systems; sharing electronic health records between jails and public clinics; increasing public health education and outreach in jails; and creating jail reentry centers that house primary medical, substance use, mental health care, and care management services that help secure housing and employment assistance^c^. In addition, courts should consider a medical diversion approach that releases low-risk pretrial detainees with chronic health conditions into the community and provides funded healthcare coverage. This approach would allow detainees to receive continuous services in the community while enabling county governments to avoid the additional expense of providing clinical services for detainees who have health coverage and a means of accessing behavioral healthcare treatment in the community.

#### Future challenges

As the healthcare landscape changes over the next several years and beyond, various challenges will include enrolling individuals involved in the criminal justice system into Medicaid, transferring large numbers of referrals from the criminal justice system to community treatment providers, creating partnerships at the system and program levels, and avoiding net-widening. Net-widening involves more stringent intervention programs that actually increase the number of individuals returned to jail or prison for violations of their terms of release. Net-widening can also occur when low-risk individuals are placed in more rigorous programs to ensure their access to needed services.

The potential barriers to treatment for criminal justice-involved populations under a Medicaid-dominated public treatment system should be identified as soon as possible, while the ACA is being implemented and related polices are being formulated. Specifically, several fundamental issues must be addressed: (1) Can plans be allowed to deny the reimbursement for care solely because a referral is from the criminal courts? (2) How can people meet the criteria for medical necessity for substance abuse treatment when leaving an incarcerative setting? (3) How does patient choice in healthcare decisions impinge on mandated treatment participation?

In 2014, the justice-involved population will likely be able to choose their own healthcare provider networks with the nation’s move toward Medicaid-managed care and with the variety of plans offered on the Marketplace. Although the right to choose is antithetical to the notion of a criminal justice mandate, as long as the criminal justice system has processes in place to recommend appropriate levels of care for each treatment episode, clients and staff will be able to choose treatment programs from a network of approved providers that supply varying levels of care. States have experimented with this process under the federal Access to Recovery Initiative (U.S. Department of Health and Human Services [DHHS], 2007). Evidence suggests that clients are more likely to complete treatment when they weigh into their own healthcare decisions (van Til et al. 2010). Collaboration between criminal justice practitioners and local providers in developing a network of care will increase the chances that both systems will meet their respective goals. Therefore, the successful implementation of healthcare reform will require the education of individuals and families involved in the criminal justice and treatment systems to help consumers become self-sufficient in accessing, maintaining, and remaining accountable for their own healthcare.

Finally, to optimize the potential of the ACA, the reality of chronic health conditions must be acknowledged and the standards of care for such conditions must be woven into the criminal justice and healthcare systems. Those responsible for the implementation of healthcare reform at state and local levels must work together to advocate and implement reforms that meet the long-term healthcare needs of individuals with chronic health conditions. Success in overcoming these philosophical and practical challenges will result in greater health and justice for millions of individuals and families, reduced crime and recidivism, and healthier and safer communities nationwide.

## Endnotes

^a^Based on 2014 Federal Poverty Level Guidelines.

^b^The Bureau of Justice Assistance defines medium-sized jails as those with an average daily population (ADP) of 500 to 999 (Stinchcomb et al. 2009). The mid-point in that range is approximately 750. Assuming that the average length of stay for a detainee is 14 days, 750 jail beds x 26 time periods per year equals an annual jail population of 19,500. A 10 percent reduction of 19,500 equals a reduction of 1,950 detainees a year. At a rate of $75/day x 14 days, the savings equated with a 1,950-detainee reduction equals $2,047,500.

^c^Medicaid benefits cannot be accessed until an individual receives healthcare services in the community and will not pay for services rendered in a correctional setting.
